# Study of the Milkability of the Mediterranean Italian Buffalo and the Tunisian Maghrebi Camel According to Parity and Lactation Stage

**DOI:** 10.3390/ani14071055

**Published:** 2024-03-29

**Authors:** Moufida Atigui, Marwa Brahmi, Pierre-Guy Marnet, Wiem Ben Salem, Maria Concetta Campagna, Antonio Borghese, Giuseppe Todde, Maria Caria, Mohamed Hammadi, Carlo Boselli

**Affiliations:** 1 Livestock and Wildlife Laboratory, Arid Regions Institute, IRESA, Medenine 4100, Tunisia; 2 Higher Institute of Agricultural Science of Chott-Mariem, Sousse 4000, Tunisia; 3 Department of Animal and Food Sciences, Institut Agro Rennes-Angers, F-35042 Rennes, France; pierre-guy.marnet@agrocampus-ouest.fr; 4 UMR SELMET, CIRAD, INRAe, Institut Agro, F-34398 Montpellier, France; 5 Livestock and Pasture Office, Tunis 1002, Tunisia; 6 Experimental Zooprophylactic Institute Lazio and Toscana Mariano Aleandri, 00178 Rome, Italycarlo.boselli@izslt.it (C.B.); 7 International Buffalo Federation, 00015 Rome, Italy; antonio.borghese@email.it; 8 Department of Agricultural Sciences, University of Sassari, 07100 Sassari, Italymariac@uniss.it (M.C.); 9 Ecole Doctorale Science, Ingénierieet Société, Université de Gabès, Gabès 6029, Tunisia

**Keywords:** milkability, milk flow, Lactocorder, Mediterranean Italian buffalo, Maghrebi camel

## Abstract

**Simple Summary:**

Milk flow kinetics recording has been proven to be a valuable tool for milkability assessment in dairy animals. In this study, milk flow curves were recorded for Mediterranean buffaloes and Maghrebi she-camels and the effect of parity and lactation stage were tested. Results revealed that both buffalo and camel species have overall good milkability characteristics, with good milk production/milking and milk flow rates. Buffaloes had higher delayed milk ejection and a high prevalence of overmilking, resulting in longer milking time compared to camels. This study highlighted the need for good management practices to achieve a good milkability in both species.

**Abstract:**

While considered as hard milkers, both buffaloes and camels are milked with equipment destined for dairy cows based on external morphological similarities with this species. This work aimed to study similarities and differences in milkability traits between Mediterranean buffaloes and Maghrebi she-camels and to evaluate the effect of parity and lactation stage. A total of 422 milk flow curves recorded with an electronic milkmeter (Lactocorder^®^) for both species were accessed. Milking characteristics including milk yield per milking, peak milk flow, average milk flow, duration of the main milking phase, duration of total milking, duration of various phases of milk flow, lag time and time to milk ejection, stripping yield, overmilking time and incidence of bimodal milk flow curves were evaluated for both species. Results showed that the values of milk yield per milking, duration of the main milking phase and duration of total milking were higher in buffaloes (3.98 ± 0.10 kg; 4.07 ± 0.11 min; 9.89 ± 0.21 min, respectively) compared to camels (3.51 ± 0.08 kg; 3.05 ± 0.09 min; 3.76 ± 0.09 min, respectively). However, camels had significantly higher peak and average milk flow (2.45 ± 0.07 kg/min and 1.16 ± 0.03 kg/min, respectively). Camels took significantly less time for milk ejection to occur. Only 15.49% of recorded curves were bimodal in buffaloes while 34.93% of bimodal curves were recorded for camels. Overmilking was significantly higher in buffaloes (3.64 ± 0.21 min vs. 0.29 ± 0.02 min). Parity and lactation stage had a significant effect on most studied milking traits suggesting the need for some particular practices with primiparous animals and animals at different levels of lactation for both species.

## 1. Introduction

In the actual context of global warming, both buffaloes (*Bubalus bubalis*) and camels (*Camelus dromedarius*) have particular interest as nonbovine dairy species that have several advantages. These species are well adapted to harsh environments and constrained climatic conditions including high temperature, high humidity, and/or severe aridity [1]. In addition, consumption of milk of both species is increasing rapidly in the last years due to their nutritional and therapeutic proprieties. Indeed, in Italy, buffalo milk has a high market value. It has a particularly high content of fat (7–8%) and is used to produce Mozzarella di Bufala Campana PDO cheese which is a Protected Designation of Origin food [2]. Similarly, recent studies on camel milk and its products indicated several biological and functional properties, including antidiabetic, cholesterol-lowering, ACE-inhibitory, hypoallergenic, antioxidant and immunomodulatory activities [3,4,5,6,7]. 

Both buffaloes and camels are considered to be difficult to be machine milked compared to cows. Indeed, both species have very limited cisternal milk [8,9,10] that can hardly be removed via the milking vacuum. The main anatomical–physiological differences between buffalo and dairy cows could be found in the mammary cistern, located in the ventral part of the gland, which functions as a milk storage area that allows the synthesis of different amounts of milk depending on its size. The buffalo cow’s udder can store 92–95% of the milk in the alveolar compartment; the rest (about 5%) is stored in the cistern. Dairy cows, by contrast, store 20% of their milk in cisternal compartment. The cisternal milk fraction is available for milking or for calf suckling before the epithelial cells contract in response to oxytocin, which triggers the expulsion of the milk. Alveolar milk, however, is only available if it is actively expelled as a result of the concentration of oxytocin in the blood during milking [11]. Also, both are very sensitive to environmental stimulus before and during milking which explains the disrupted milk ejections observed for buffaloes [8,12] and camels [13,14]. In addition, because of external udder morphology similarities with bovine species, these animals are usually milked with the same milking equipment but with different settings to respect the physiological needs of each species [14,15,16]. Evaluation of milkability characteristics in nonbovine dairy animals has been reported to improve milking efficiency, reduce working vacuum on-time on empty teats [2,17,18] and could be used to select suitable animals with similar milkability traits for an optimal milking process [2,19]. In particular, milk flow profiles and milking characteristics recorded using milk meters such as the Lactocorder^®^ (WMB, Balgach, Switzerland), have been successfully used to evaluate the efficiency of the milking process for several species including cattle [20,21,22], small ruminants [23,24,25], buffaloes [2,15,17,19,26] and camels [13,16,18,27,28]. Furthermore, it has been reported that milk flow patterns are generally typical for each animal [27,29] and therefore could be a useful tool to diagnose any modification or disturbance of the milking routine that could potentially cause uncomfortable milking or disrupt milk ejection. 

Besides teat anatomy [18,30], milking machine settings [16,31], milking routine [12,13,17,26], milking environment [12,13,27] and health status [2,31], parity and lactation stage significantly affect milk flow traits suggesting a particular treatment for primiparous animals or animalsin advancedstages of lactation [20,27,32]. Several studies reported that milk yield and milk flow rate are higher in Mediterranean Italian buffaloes at the third or fourth lactation [33,34]. Di Palo et al. [35] found a higher milk yield and decline phase in pluriparous than primiparous buffaloes. Similarly, multiparous camels also produced significantly more milk than the primiparous ones [18]. Similarly, studies reported a significant effect of parity on most of the milkability traits including milk yield, milk flow rate, milking time and bimodality in camels [16,27]. Primiparous camels, being more fearful than multiparous ones, require more attention during milking to avoid disrupting the milk ejection reflex, which, consequently, interrupts milk flow. Additionally, milk flow characteristics are significantly influenced by lactation stage. In dromedaries, time-until-milk-ejection is shorter in the early and middle stage of lactation as compared with late-lactation [18,28]. A faster milk flow is observed when the milk yield is higher; thus, milk flow rate was higher during the peak of lactation [36]. Bimodal curves were mostly recorded during early- and middle-lactation for both species [18,28,32]. In studies on Mediterranean buffaloes, Bava et al. [32] reported that the delay time of milk ejection remained very long and increased significantly with the increase in the lactation phase, while peak flow rate (PFR) and average flow rate (AFR) decreased significantly with the increase in the lactation stage. According to the lactation phase, camels produced a higher daily milk yield (DMY) at the beginning of lactation than at middle and late lactation; also, the PFR and AFR were also significantly influenced by the milk flow curve type and the parity [18].

In dairy animals, delayed milk ejection causes bimodal milk flow curves [24,36] with separate emission of cisternal and alveolar milk fractions, while a unique emission peak refers to an optimal stimulation and physiological oxytocin release. However, for anatomical and physiological reasons, animals with tight streak canals, such as buffaloes [17,30,37] or within some camels needing a high vacuum to open teat sphincters [38], a milk flow pattern with a delayed single emission peak is frequently observed. This could be explained by a tight closure of the teat canal that requires tactile stimulation and increased intramammary pressure to overcome the teat barrier rather than a higher vacuum level. A long delay between cluster attachment and milk emission peak could likely be due to suboptimal prestimulation of the teats, leading to a delayed release of oxytocin into the bloodstream for both buffaloes and dromedary species. Therefore, the aim of this study was to test a hypothesis to investigate the similarities and differences in milkability traits between Mediterranean Italian buffaloes and Maghrebi she-camels, and to evaluate the effect of parity and lactation stage. 

## 2. Materials and Methods

### 2.1. Animals and Milking Routine

Buffaloes: Clinically healthy buffaloes in different parities (1–7) and lactation stages (12–328 Days in Milk (DIM)) from two private farms located in Latina (Italy), were used for this study. On farm A, buffaloes were milked in a low-level herringbone (2 × 5) milking parlour (DeLaval, Tumba, Sweden). On the second farm (B) the milking system was a 2 × 28 parallel parlour (Harmony Plus, DeLaval, Tumba, Sweden) with a low-level milking system equipped with automatic cluster removers and an electronic herd management system (Alpro system, DeLaval). Automatic cluster detachment has been disabled during the milking sessions. In both farms, the pulsation rate was 60 cycles/min, and the pulsation ratio was 60%; during the experimental milking, the vacuum level was set at 49 kPa.

Camels: Clinically healthy camels in different parities (1–6) and lactation stages (49–335 DIM) belonging to the experimental farm of the Arid Regions Institute (IRA, Chenchou, Tunisia) were used. Animals were maintained exclusively in the farm in a loose-stall housing system. Camels were milked in a restraining stall using a portable milking machine (Model AM/T115, Agromilk, 42020S. Polo d’Enza, Reggio Emilia, Italy) equipped with DeLaval Clusters. Machine milking was set at 60 cycles/min, a 60% pulsation ratio and a 48 kPa vacuum level.

For both species, the milking routine included teat cleaning and manually ejecting the first milk jets from the teats followed by attachment of the milking unit and manual removal of the milking unit by the milker at the end of milking. Stripping is performed by manually pulling the milking cluster after the milk flow decreased to less than 0.1 kg/min, before vacuum shut off. No concentrates were given to the animals during milking.

### 2.2. Milk Flow Measurement

A total of 422 milk flow curves were recorded for both species (213 for buffaloes and 209 for camels) using a milk flow meter, the Lactocorder (WMB, Balgach, Switzerland). The milk meter started recording information at the first teat cup attachment and finished when teat cups were detached.

The evaluation of milk flow curves was conducted with specific available software (LactoPro; WMB, version 5.2.0) to evaluate 13 milk flow traits for both species including the following: 

MMY (Machine Milk Yield, kg): milk yield per head per milking from the beginning to the end of the mechanical milking;

Lag time (min): time elapsed from the beginning of measurement until a 0.25 kg/min threshold in the milk flow was reached;

EMT (Effective Milking Time, min): time elapsed from time when milk flow rate is >0.25 kg/min until milk flow drops below 0.10 kg/min; 

TMT (Total Milking Time, min): time from milking cluster attachment till removal, including stripping and overmilking times if they are present;

APT (Ascendant Phase Time, min): time elapsed from milk flow <0.25 kg/min at the beginning of milking to the top of the plateau phase. 

PPT (Plateau Phase Time, min): duration of plateau phase from the vertex of the incline phase to the vertex of the decline phase;

DPT (Decline Phase Time, min): time recorded from the end of the PPT until the flow rate drops below 0.10 kg/min;

tMBG (time of Mechanical Overmilking, min): if present, tMBG is recorded at the end of the DPT, with milk flow less than 0.10 kg/min;

PFR (Peak Flow Rate, kg/min): highest flow rate in the main milking process that lasted at least 22.4 s;

AFR (Average Flow Rate, kg/min): average milk flow during the EMT;

tPFR (time of ¨Peak Flow Rate, min): time when milk flow reached its peak;

MNG (Stripping Yield, kg): milk yield recorded during the post milking phase after the EMT ended and milk flow increased again to more than 0.30 kg/min and did not exceed 1 kg/min or 50% of PFR;

BIMO (Bimodality, %): bimodality of milk flow, which resulted from the interruption of the flow at the start of milking when cisternal milk was finished and alveolar milk was not yet available;

TME (Time to Milk Ejection, min): TME is visually determined via the observation of teat swelling in camels and estimated on the milk flow curve generated with the Lactocorder as the onset of the main fraction of milk.

### 2.3. Statistical Analysis

All data are presented as arithmetic mean values ± SEM. Data were statistically analysed using a SAS program (SAS version 9.4, SAS Inst. Inc., Cary, NC, USA). Basic statistics were calculated via the UNIVARIATE procedure. The MIXED procedure was used for main evaluation according to the following model:

Y_ijklm_ = μ + Sp_i_ + P_j_ +LS_k_ + F_l_ + (Sp × P)_ij_ + (Sp × LS)_ik_ + (Sp × F)_il_ + A_m_ + e_ijklm_ (1)

where Y_ijklm_ is the individual observation of measured traits, including MMY (kg), TME (min), Lag time (min), TMT (min), EMT (min), AFR (kg/min), PFR (kg/min), APT (min), PPT (min), DPT (min), tMBG (min), and MNG (kg); μ is the overall mean, Sp_i_ is the fixed effect of species (i = 1, 2), P_j_ is fixed effect of parity (j = 1, 2), LS_k_ is the fixed effect of lactation stage (j = 1 to 3), A_m_ is the random effect of the m^th^ animal and e_ijkl_ is the random residual error.

A multiple comparison of least square means (LSM) for the fixed effects was performed using the PDIFF (pairwise difference by least significant difference) test (*p* < 0.05, otherwise stated). The χ^2^ test was used to evaluate group differences in the bimodality trait. Pearson correlation coefficients among milking traits according to the corresponding species were calculated using the correlation procedure (PROC CORR). A linear regression was performed using PROC REG for each species individually to evaluate the relationship between lag time and TME. 

## 3. Results

### 3.1. Milkability Traits of Mediterranean Italian Buffaloes and Maghrebi She-Camels

Basic statistics for the monitored milkability parameters of the tested buffalo cows and she-camels are presented in Table 1. 

The results of this study showed highly significant differences for all milkability traits, except for the MNG. Buffaloes had significantly higher MMY, longer lag time, TME and effective and total milking time, while camels had significantly higher maximum and mean milk flow rates and a higher incidence of bimodality. Milk yield per milking varied significantly for both species.

**Figure 1:**
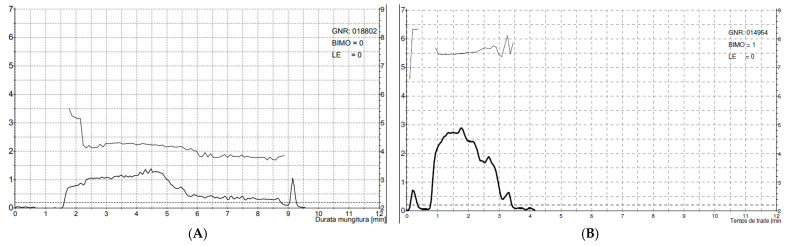
Typical milk flow profile for monitored buffaloes (**A**) and camels (**B**).

MMY ranged from 0.75 to 7.78 kg and 1.53 to 7.41 kg for buffaloes and camels, respectively. The milk flow curves showed a significantly longer PPT in buffaloes, while the APT and DPT were not significantly different between the two species (Figure 1). The PPT:DPT ratio was 71.42% and 19.12% for buffaloes and camels, respectively.

The Pearson’s correlations between the main milkability traits of the Maghrebi she-camels and the Mediterranean Italian buffalo cows are shown in Table 2. 

Pearson correlation coefficients indicated that MMY was positively and strongly related to PFR (r = 0.60; *p* < 0.0001 and r = 0.78; *p* < 0.0001), AFR (r = 0.59; *p* < 0.0001 and r = 0.62; *p* < 0.0001) and EMT (r = 0.50; *p* < 0.0001 and r = 0.30; *p* < 0.0001), respectively, for buffaloes and camels; while MMY had a positive weak correlation with PPT in buffaloes (r = 0.26; *p* < 0.0001), it was negatively related in camels (r = −0.22; *p* = 0.001). Also, results showed that MMY was strongly correlated with TMT in camels (r = 0.64; *p* < 0.0001) while in buffaloes these two traits were not correlated (*p* = 0.90). Further, MMY was negatively and weakly correlated with lag time and TME (r = −0.22; *p* = 0.008 and r = −0.27; *p* < 0.0001, respectively) in buffalo species and were not correlated in camels. Time of incline phase revealed a positive correlation with bimodality of the milk flow curves for both species.

Interestingly, PFR was moderately and negatively correlated with PPT (r = −0.40; *p* < 0.0001) in camels. A positive correlation between TMT, overmilking time and TME was detected in buffaloes (r = 0.78; *p* < 0.0001 and r = 0.34; *p* < 0.0001, respectively), while only TME was strongly correlated with TMT (r = 0.64; *p* < 0.0001) in camels. 

**Figure 2:**
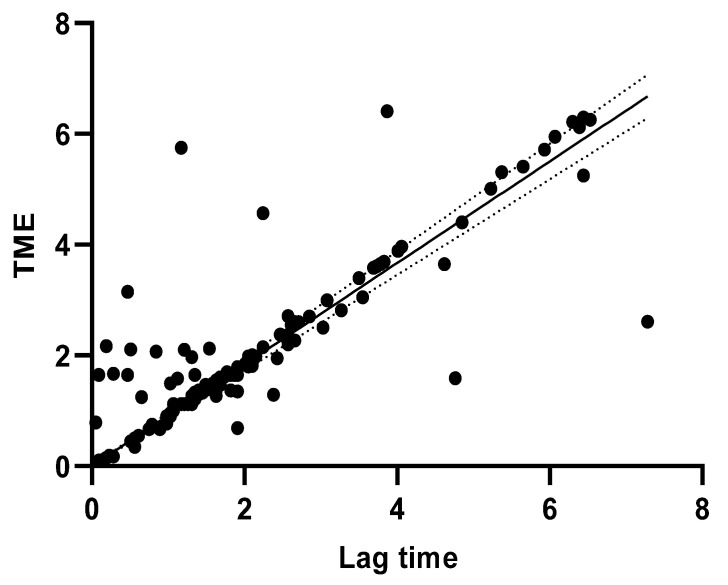
Linear relationship between lag time and TME in buffaloes, R^2^ = 0.71 (Graph Pad program).

The Pearson correlation coefficient between TME and lag time was strong in buffaloes (r = 0.84; *p* < 0.0001) and moderate in camels (r = 0.31; *p* < 0.0001). The estimated linear regression between these two traits showed a high coefficient of determination for buffaloes (R^2^ = 0.71) (Figure 2) compared to camels (R^2^ = 0.1). 

### 3.2. Effect of Parity on Milkability Traits 

The effect of parity on milk yield and milk flow traits is shown in Table 3. Parity had significant effect on all parameters studied except for MNG for both species. MMY was similar in multiparous buffaloes and camels (*p* = 0.21), followed by primiparous buffaloes and then primiparous camels, which produced significantly (*p* < 0.0001) less milk.

Within the same species, TME, lag time, EMT and TMT did not differ significantly and were significantly shorter (*p* < 0.0001) in primiparous and multiparous camels compared to buffalo at both parities.

AFR was significantly higher in multiparous camels while primiparous camels had an AFR within the same range of buffaloes of different parities. The highest (*p* < 0.0001) PFR was found in multiparous camels, while the lowest was found in primiparous and multiparous buffaloes (*p* = 0.07).

Overmilking time (tMBG) was significantly (*p* < 0.0001) longer in buffaloes with no difference between primiparous and multiparous ones. The highest percentage of bimodal curves was found in camels, in multiparous compared to primiparous ones (37.82% vs. 26.42%, *p* < 0.05), while buffaloes showed significantly lower bimodality in primiparous and multiparous ones (11.27% vs. 17.61%; *p* < 0.05).

### 3.3. Effect of Lactation Stage on Milkability Traits

As shown in Table 4, in both species, the lowest MMY was recorded at the late stage of lactation. Moreover, buffalo cows produced significantly more milk at early- (*p* < 0.0001) and mid-lactation (*p* < 0.05) than dromedary dams. TME and lag time increased significantly with advancing lactation stage in buffaloes, while lag time remained stable in camels. EMT decreased significantly during lactation for both species. TMT evolved in line with EMT in camels (*p* < 0.05), while it remained stable throughout lactation in buffaloes (*p* > 0.44). Time to reach peak flow was long and did not vary significantly across lactation stage in buffalo species. AFR and PFR were significantly higher at early-lactation for buffaloes while they reached their maximum at mid-lactation in camels. In camels, PFR reached its maximum at late- and mid-lactation compared to early-lactation. A shorter PPT was recorded at late-lactation in buffaloes compared to early- and mid-lactation, while it remained steady along lactation stages in camels. Within the same species, tMBG did not differ significantly throughout lactation. More than half of the curves were bimodal at early-lactation in camels (51.47%) and decreased significantly (χ^2^ = 20.16; *p* < 0.0001) with increasing lactation stage. By contrast, bimodality was highest in mid-lactation compared to early- and late-lactation in buffaloes (16.22%; 15.38% and 14.58%, respectively).

## 4. Discussion

Milkability traits have been previously studied in buffaloes and camels and it has been shown that the pattern of milk secretion is typical of each species [27,39,40], as confirmed by the results obtained in our study, which are similar to those reported in the literature for buffaloes [2,17,26,29,32,35,39] and camels [13,18,27,28,41]. 

Findings related to milk yield revealed that Mediterranean Italian buffaloes have higher milk production than Tunisian Maghrebi Camels. In fact, the Mediterranean Italian buffalo is a specialised dairy breed recognised by the ANASB in Italy and subject to an official genetic selection programme for dairy traits [30], whereas the Maghrebi Camel has not yet been subject to any selection programme based on dairy traits or milkability.

A wide range of milk yields has been reported for camel breeds in the literature, depending on different environmental conditions. In machine-milked camels under intensive management, milk production per milking ranged from 2 to 7 kg [10,27]. Abdalla et al. [42] suggested that the wide range of milk production in Maghrebian camels indicates a high potential for the development of milk production traits in a long-term selection program. Although MMY was higher in buffaloes, PFR and AFR were significantly lower in buffaloes than in camels.

**Figure 3:**
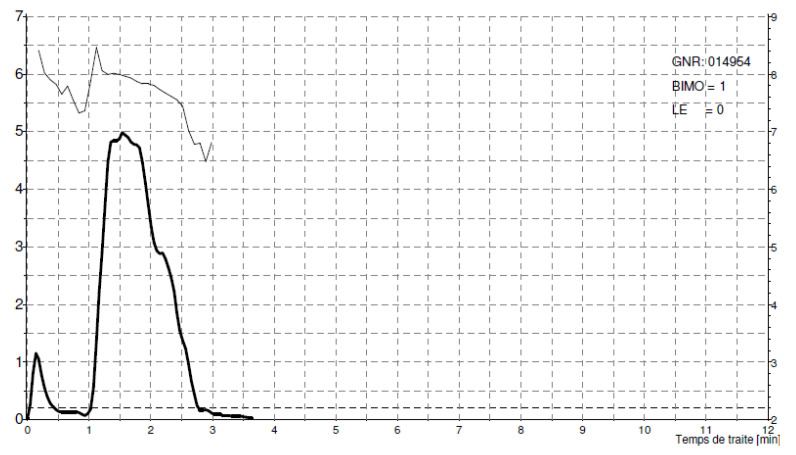
Milk flow pattern in camels with a high milk flow rate and prompt plateau phase.

As shown in Figure 1, buffaloes are characterised by limited flow and long TMT, which are basically associated with a long and tight teat streak canal. Teat canal length (TCL) recorded in the Mediterranean Italian breed ranged, on average, between 21.78 ± 0.74 mm and 23.6 ± 1.1 mm [30,37]. On the contrary, in camels, an immediate and short PPT and a very high PFR (Figure 3) were associated with a lower resistance of the teat sphincter and/or a larger diameter of the teat canal when fully open, allowing rapid emptying of the udder [27]. The TCL recorded in camels appears to be shorter than the range reported for buffaloes; in fact, recent studies conducted on the Mediterranean Italian breed have reported a mean TCL from 1.29 ± 0.62 cm to 1.47 ± 0.20 cm [38,43], which could explain a higher PFR in Maghrebi camels. Such differences could explain longer the time to reach peak flow rate (tPFR) and longer PPT in buffaloes. Other research has shown that TCL significantly influences milk flow characteristics even in dairy cows, with a negative correlation observed between TCL and mainly milk flow traits as MYM, AFR, PFR and somatic cell score [44,45]. Animals with faster milk flow tend to have shorter teat canals, which offer less resistance to milk flow, highlighting the role of teat anatomy, particularly canal length, in determining milking efficiency and emptying of the udder.

The EMT was one minute longer in buffaloes while the TMT was six-times longer. Overall, the TMT recorded for buffaloes in this study was within the range of previous studies conducted on this breed [2,33]. Caria et al. [15] tested the effect of different vacuum levels (40–52 kPa) on milkability traits. Although higher vacuum levels reduced the EMT, the TMT remained long (about 10 min), so the authors recommended the use of lower vacuum levels. Similarly, the effect of vacuum level on milking traits has been tested in camels and showed the need for high vacuum levels to completely empty the udder and shorten milking time [16,46].

However, both species are characterised by reduced udder cistern size [8,9,17] and delayed milk ejection, which took an average of 1.02 ± 0.06 min and 2.06 ± 0.12 min in camels and buffaloes, respectively. This means that the teat canal is often exposed to a long period of vacuum without milk ejection, which can damage the delicate teat tissue and compromise the teat barrier, increasing the risk of subclinical mastitis. A positive relationship between increasing working vacuum level and milk somatic cell count (SCC) has been found in buffaloes [2,47,48] and camels [43]; also, higher pulsation frequency and pulsation ratio have been found to increase both milk yield and SCC [49].

However, some studies have reported that buffalo and camel species are more tolerant of higher milking machine vacuum settings than cattle. Ayadi et al. [46] found that vacuum levels up to 50 kPa were not associated with subclinical mastitis and the camels tested had a California Mastitis Test score <1 and an average of 387 × 10^3^ cells/mL in milk. In buffaloes, a high pulse ratio was associated with increased milk production without any effect on the SCC score, according to Matera et al. [48].

**Figure 4:**
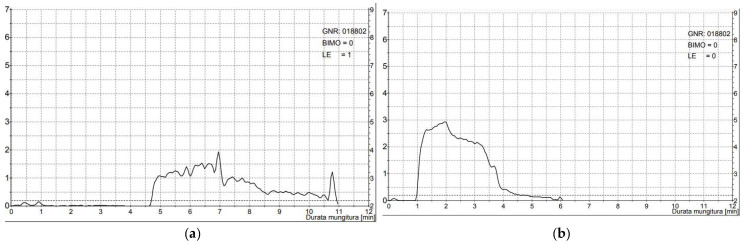
Delayed (**a**) and normal (**b**) milk ejection in buffalo species.

Manual stimulation of teats before milking is crucial in different dairy species, to start milking ejection and to reduce the combined effect of high working vacuum levels with the absence of milk on teats [13,17,26,37,41] as shown in Figure 4.

Bimodal milk emission curves recorded in camels were significantly higher than in buffaloes. A bimodal milk flow curve may be the result of inadequate or absent stimulation prior to cluster attachment or a short latency period between stimulation and milking cluster attachment. The lower percentage of bimodal curves recorded in buffaloes may be related to the narrower teat sphincters, as previously explained, which require greater intramammary pressure to overcome the teat barrier and allow milk to escape. Among the many factors that influence the kinetics of milk ejection, bimodality has been widely used to evaluate the quality of the premilking routine and the efficiency of udder stimulation in bovine species [20,36,50]. This is partially true for the buffalo species, as a vacuum level of up to 45 kPa is generally ineffective in opening the teat sphincter, unless alveolar milk ejection has occurred [37]. Therefore, to evaluate the milking routine and its effectiveness in stimulating the udder, we can consider the delay time as the most reliable characteristic reflecting the milk ejection reflex (Figure 2).

MNG was very low for both species (less than 0.1 kg), suggesting that stripping can be eliminated from the milking routine with negligible loss of production and reduced labour time. High values of overmilking (tMBG) were recorded in the buffalo species, probably related to the deactivation of automatic cluster detachment, while in the experimental camel farm, rapid teat removal was recommended. 

Longer milking duration with increased vacuum in the mouthpiece chamber during low milk flows at the end of milking could result in severe consequences on teat conditions measured as increased teat tissue thickness [51]. With lower milk yield in front teats in both buffaloes [17] and camels [52], the expected effect of overmilking should be higher on these teats. Vierbauch et al. [53] reported significantly higher vacuum levels in the mouthpiece chamber at the rear teats during milking and overmilking whereas damage was only detected on front teats. Thus, the use of automated cluster removal is recommended to reduce the risk of overmilking. 

In this study, reduced milk yield was associated with delayed milk ejection only in buffaloes. Delayed milk ejection is mainly caused by unsatisfied physiological requirements of the animals caused by insufficient blood oxytocin concentration just before milking and during milking or improper timing of milking unit attachment. 

In dairy cows, delayed milk ejection has been associated with reduced milking efficiency, reduced milk yield and impaired teat and udder health [54]. As expected, MMY was positively related to PFR, AFR and EMT in both species. The same trend was reported in a previous study in camels [27] and in dairy cows [55]. In this study, we found a negative correlation between PFR and PPT in camels.

As mentioned above, several milk flow profiles recorded in camels are characterised as type 1 milk kinetics, where the milk flow is never restrained during milking, resulting in higher peak flow values and a very short PPT as described by Atigui et al. [27].

In the present study, variations related to parity and stage of lactation were identified for several milkability traits in both species. The results show that MMY was higher in buffaloes and multiparous camels than in primiparous ones. These results were consistent with studies conducted in buffaloes [35], camels [18,27,28] and dairy cows [20,55,56]. Within the same species, our results showed no significant differences in TME, lag time, EMT and TMT between primiparous and multiparous animals. However, the AFR and PFR were significantly higher in multiparous camels, while they remained in the same range for buffaloes of different parities. This is in partial agreement with the results reported for buffaloes by Di Palo et al. [35] and for camels by Atigui et al. [27].

Differences between animals of different parities could be related to a changing teat anatomy with advancing parity [17,27,30,57,58]. Milking characteristics varied according to the stage of lactation due to changes in milk yield with advancing lactation. In buffaloes, EMT decreased in late-lactation with decreasing milk yield, whereas TMT did not vary with increasing DIM, suggesting that the effect of the machine on the time of vacuum on empty teats was higher at this stage. In fact, lag time and overmilking were significantly higher in late-lactation and represented about 70% of the total milking time.

In dairy buffaloes, several authors [17,59,60] had related the main anatomical features of the buffalo teat to milk flow. Napolitano et al. [60] reported that the milk flow curve in the buffalo dairy cow has several stages, from initial milk ejection through stimulation to constant milk flow, indicating the need to pay special attention to the premilking routine, as delays in milk ejection tend to reduce milking capacity, which may also be influenced by the teat anatomy of individual dairy buffaloes, stage of lactation, parity, udder conformation and breed. AFR and PFR decreased with lactation stage in buffaloes, whereas they were significantly higher in camels in mid-lactation. Borghese et al. [39] found that the time to milk ejection was shorter in early- and mid-lactation compared to late-lactation. AFR and PFR were higher when yield was higher. These authors suggested that if buffaloes were carefully selected for milk yield and milkability, these traits could be improved. Bimodal curves were highest in camels in early-lactation, in agreement with the results reported by Atigui et al. [27]. They recorded 70% of bimodal curves in early-lactation, compared to only 8–9% in late-lactation. This is due to the decrease in the cisternal milk fraction as lactation progresses and the low level of udder filling [10].

Finally, although this study is the first to compare milking characteristics between two different species, it has some limitations that need to be discussed. Even though the milking routine and the milking machine settings, in particular the working vacuum level, were standardized, it was not possible to standardize the environmental factors, such as the experimental site (different geographical location) and the farm management, which could have influenced the results obtained. In addition, the Mediterranean buffalo breed used in the study was the result of more than half a century of genetic selection, whereas the camels used in this study belonged to an experimental herd where animals were selected for their milking characteristics. Lastly, this study provides useful guidance on the effect of parity and lactation on milk yield in both species, but milk flow patterns may be influenced by several other environmental factors. Future studies on this topic should include larger samples of animals to allow for the analysis of non-genetic factors on milk flow characteristics.

## 5. Conclusions

Based on the results of the present study, it can be concluded that both buffalo and camel species have overall good milkability characteristics. Comparison between the two species showed delayed milk ejection and a high prevalence of overmilking in buffaloes, which could explain the longer milking time compared to camels. On the other hand, camels showed a higher prevalence of bimodal milk flow kinetics. Primiparous animals had lower milk production and percentage of bimodal curves than multiparous animals in both species. Primiparous camels had a lower milk yield than multiparous camels, while in buffaloes, parity had no effect on milk yield. Lactation stage significantly influenced some milkability traits. Milk yield and effective milking time decreased during lactation in both species. Milk ejection time was longer in the buffaloes at the end of lactation, whereas it was longer in the camel at the beginning of lactation. Bimodality decreased significantly with stage of lactation increase in the camels, whereas it remained constant in the buffaloes. This study suggests the need for prestimulation to reduce milk ejection time in buffaloes and bimodality in camels. The use of automatic cluster removal is also recommended, particularly in buffalo species. 

## Figures and Tables

**Table 1:** Basic statistics for milkability traits tested for buffaloes and camels.

	Buffaloes (213)	Camels (209)
MMY, kg	3.99 ± 0.10 ^a^	3.52 ± 0.08 ^b^
TME, min	2.06 ± 0.12 ^a^	1.02 ± 0.06 ^b^
Lag time, min	2.12 ± 0.11 ^a^	0.27 ± 0.03 ^b^
EMT, min	4.07 ± 0.11 ^a^	3.05 ± 0.09 ^b^
TMT, min	9.89 ± 0.21 ^a^	3.76 ± 0.09 ^b^
AFR, kg/min	0.93 ± 0.02 ^b^	1.16 ± 0.03 ^a^
PFR, kg/min	1.50 ± 0.04 ^b^	2.45 ± 0.07 ^a^
tPFR, min	3.17 ± 0.12 ^a^	1.25 ± 0.07 ^b^
BIMO, %	15.49 ^b^	34.93 ^a^
APT, min	0.47 ± 0.05	0.65 ± 0.05
PPT, min	1.50 ± 0.08 ^a^	0.39 ± 0.05 ^b^
DPT, min	2.10 ± 0.10	2.04 ± 0.08
MNG, kg	0.10 ± 0.06	0.07 ± 0.01
tMBG, min	3.64 ± 0.21 ^a^	0.29 ± 0.02 ^b^

MMY: machine milk yield; TME: time to milk ejection; Lag time: time to reach 0.25 kg/min threshold; EMT: effective milking time; TMT: total milking time; AFR: average flow rate; PFR: peak flow rate; tPFR: time of peak flow rate; BIMO: bimodality; APT: ascending phase time; PPT: plateau phase time; DPT: decline phase time; MNG: stripping yield; and tMBG: overmilking time. ^a,b^: Means in the same line with a different superscript letter are significantly different (*p* < 0.05)

**Table 2:** Pearson’s coefficient of correlation of main milkability traits according to species.

Traits	MMY	TME	Lag Time	EMT	TMT	AFR	PFR	tPFR	PPT	DPT	APT	MNG
Buffaloes												
TME	−0.23 **	1										
Lag time	−0.27 **	0.84 ***	1									
EMT	0.50 ***	−0.04	−0.23 **	1								
TMT	0.008	0.34 ***	0.32 ***	0.12	1							
AFR	0.59 ***	−0.16	−0.14	−0.23 **	−0.12	1						
PFR	0.60 ***	−0.18 *	−0.20 *	−0.06	−0.15	0.86 ***	1					
tPFR	−0.07	0.75 ***	0.76 ***	0.20 *	0.34 ***	−0.21 *	−0.23 **	1				
PPT	0.26 **	−0.02	−0.03	0.42 ***	0.16	−0.14	−0.30 ***	0.22 *	1			
DPT	0.31 ***	−0.10	−0.18 *	0.63 ***	−0.01	−0.17 *	0.07	−0.04	−0.27 **	1		
APT	0.18 *	0.09	−0.10	0.37 ***	0.05	0.01	0.16	0.20 *	−0.02	−0.11	1	
MNG	−0.03	−0.09	−0.01	0.11	−0.03	−0.10	−0.09	−0.04	−0.02	0.17 *	−0.11	1
tMBG	−0.12	−0.03	−0.07	−0.28 **	0.78 ***	0.06	−0.12	−0.17	0.06	−0.25 **	−0.06	−0.06
Camels												
TME	0.06	1										
Lag time	−0.11	0.31 ***	1									
EMT	0.30 ***	0.57 ***	−0.13	1								
TMT	0.25 **	0.64 ***	0.24 **	0.82 ***	1							
AFR	0.62 ***	−0.24 **	0.01	−0.46 ***	−0.36 ***	1						
PFR	0.78 ***	−0.09	−0.09	−0.08	−0.04	0.80 ***	1					
tPFR	0.23 **	0.73 ***	0.39 ***	0.60 ***	0.70 ***	−0.19 *	0.03	1				
PPT	−0.22 *	−0.06	−0.05	−0.09	−0.16	−0.23 **	−0.40 ***	−0.09	1			
DPT	0.09	0.38 ***	−0.06	0.80 ***	0.68 ***	−0.44 ***	−0.17 *	0.29 **	−0.29 **	1		
APT	0.47 ***	0.47 ***	−0.10	0.57 ***	0.48 ***	0.42 ***	0.32 ***	0.67 ***	−0.25 **	0.07	1	
MNG	−0.02	−0.06	−0.11	−0.12	−0.08	−0.14	−0.08	−0.08	0.07	−0.10	−0.09	1
tMBG	0.06	−0.02	0.11	−0.15	0.23 **	0.10	0.21 *	0.01	−0.01	−0.14	−0.15	−0.37 ***

MMY: machine milk yield; TME: time to milk ejection; Lag time: time to reach 0.25 kg/min threshold; EMT: effective milking time; TMT: total milking time; AFR: average flow rate; PFR: peak flow rate; tPFR: time of peak flow rate; APT: ascending phase time; PPT: plateau phase time; DPT: decline phase time; MNG: stripping yield; and tMBG: overmilking time. (* *p* < 0.05; ** *p* < 0.001; *** *p* < 0.0001).

**Table 3:** Effect of parity on milk yield and milk flow traits in buffaloes and camels.

	Buffaloes		Camels	
	Primiparous (71)	Multiparous (142)	Primiparous (53)	Multiparous (156)
MMY, kg	3.79 ± 0.17 ^b^	4.04 ± 0.13 ^a^	2.68 ± 0.10 ^c^	3.88 ± 0.09 ^ab^
TME, min	2.13 ± 0.25 ^a^	2.03 ± 0.14 ^a^	1.12 ± 0.15 ^b^	0.98 ± 0.05 ^b^
Lag time, min	2.19 ± 0.19 ^a^	2.07 ± 0.13 ^a^	0.35 ± 0.10 ^b^	0.25 ± 0.03 ^b^
EMT, min	4.02 ± 0.18 ^a^	4.10 ± 0.14 ^a^	3.14 ± 0.21 ^b^	3.02 ± 0.09 ^b^
TMT, min	9.48 ± 0.35 ^a^	10.10 ± 0.26 ^a^	3.73 ± 0.23 ^b^	3.77 ± 0.09 ^b^
AFR, kg/min	0.90 ± 0.04 ^b^	0.94 ± 0.03 ^b^	0.86 ± 0.03 ^b^	1.27 ± 0.03 ^a^
PFR, kg/min	1.42 ± 0.06 ^c^	1.54 ± 0.05 ^c^	1.67 ± 0.07 ^b^	2.71 ± 0.07 ^a^
tPFR, min	3.30 ± 0.21 ^a^	3.09 ± 0.15 ^a^	1.26 ± 0.17 ^b^	1.25 ± 0.86 ^b^
BIMO, %	11.27 ^d^	17.61 ^c^	26.42 ^b^	37.82 ^a^
APT, min	0.36 ± 0.05 ^b^	0.52 ± 0.08 ^ab^	0.43 ± 0.08 ^ab^	0.73 ± 0.06 ^a^
PPT, min	1.61 ± 0.14 ^a^	1.44 ± 0.09 ^a^	0.45 ± 0.06 ^b^	0.36 ± 0.03 ^b^
DPT, min	2.06 ± 0.09 ^ab^	2.13 ± 0.11 ^ab^	2.33 ± 0.23 ^a^	1.93 ± 0.07 ^b^
MNG, kg	0.04 ± 0.01	0.13 ± 0.08	0.08 ± 0.01	0.07 ± 0.01
tMBG, min	3.21 ± 0.31 ^a^	3.85 ± 0.27 ^a^	0.14 ± 0.03 ^b^	0.33 ± 0.03 ^b^

MMY: machine milk yield; TME: time to milk ejection; Lag time: time to reach 0.25 kg/min threshold; EMT: effective milking time; TMT: total milking time; AFR: average flow rate; PFR: peak flow rate; tPFR: time of peak flow rate; BIMO: bimodality; APT: ascending phase time; PPT: plateau phase time; DPT: decline phase time; MNG: stripping yield; tMBG: overmilking time. ^a,b,c,d^: Means in the same line with a different superscript letter are significantly different (*p* < 0·05)

**Table 4:** Effect of lactation stage on milk yield and milk flow traits in buffaloes and camels.

	**Buffaloes**			**Camels**		
	**Early (91)**	**Mid (74)**	**Late (48)**	**Early (68)**	**Mid (95)**	**Late (46)**
MMY, kg	4.90 ± 0.13 ^a^	3.89 ± 0.13 ^b^	2.41 ± 0.10 ^d^	3.76 ± 0.14 ^c^	3.64 ± 0.12 ^c^	3.01 ± 0.13 ^d^
TME, min	1.78 ± 0.16 ^ab^	1.13 ± 0.22 ^b^	2.44 ± 0.27 ^a^	1.13 ± 0.12 ^b^	0.91 ± 0.08 ^c^	1.08 ± 0.07 ^bc^
Lagtime, min	1.86 ± 0.14 ^b^	2.03 ± 0.20 ^b^	2.73 ± 0.26 ^a^	0.33 ± 0.08 ^c^	0.20 ± 0.03 ^c^	0.33 ± 0.06 ^c^
EMT, min	4.55 ± 0.16 ^a^	4.27 ± 0.21 ^a^	2.87 ± 0.14 ^c^	3.48 ± 0.17 ^b^	3.05 ± 0.12 ^b^	2.42 ± 0.10 ^d^
TMT, min	9.77 ± 0.31 ^a^	10.09 ± 0.37 ^a^	9.82 ± 0.43 ^a^	4.24 ± 0.18 ^b^	3.68 ± 0.12 ^bc^	3.21 ± 0.14 ^c^
AFR, kg/min	1.06 ± 0.04 ^b^	0.91 ± 0.04 ^c^	0.72 ± 0.03 ^d^	1.09 ± 0.05 ^b^	1.20 ± 0.04 ^a^	1.18 ± 0.06 ^ab^
PFR, kg/min	1.72 ± 0.06 ^d^	1.46 ± 0.06 ^e^	1.16 ± 0.04 ^f^	2.40 ± 0.10 ^b^	2.61 ± 0.11 ^a^	2.17 ± 0.10 ^c^
tPFR, min	2.99 ± 0.17 ^a^	3.29 ± 0.24 ^a^	3.28 ± 0.25 ^a^	1.57 ± 0.14 ^b^	1.12 ± 0.09 ^c^	1.05 ± 0.10 ^c^
BIMO, %	15.38 ^d^	16.22 ^c^	14.58 ^d^	51.47 ^a^	32.98 ^b^	15.56 ^c^
APT, min	0.56 ± 0.09 ^bc^	0.46 ± 0.11 ^c^	0.30 ± 0.05 ^c^	0.85 ± 0.11 ^a^	0.64 ± 0.08 ^ab^	0.38 ± 0.06 ^c^
PPT, min	1.68 ± 0.12 ^a^	1.62 ± 0.14 ^a^	0.97 ± 0.11 ^b^	0.32 ± 0.17 ^c^	0.37 ± 0.03 ^c^	0.52 ± 0.06 ^c^
DPT, min	2.32 ± 0.15 ^a^	2.18 ± 0.19 ^a^	1.60 ± 0.12 ^bc^	2.32 ± 0.17 ^a^	2.07 ± 0.10 ^ab^	1.52 ± 0.10 ^c^
MNG, kg	0.05 ± 0.02	0.21 ± 0.16	0.02 ± 0.01	0.04 ± 0.01	0.05 ± 0.07	0.15 ± 0.03
tMBG, min	3.92 ± 0.33 ^a^	3.72 ± 0.38 ^a^	4.17 ± 0.37 ^a^	0.30 ± 0.04 ^b^	0.30 ± 0.03 ^b^	0.22 ± 0.05 ^b^

MMY: machine milk yield; TME: time to milk ejection; Lag time: time to reach 0.25 kg/min threshold; EMT: effective milking time; TMT: total milking time; AFR: average flow rate; PFR: peak flow rate; tPFR: time of peak flow rate; BIMO: bimodality; APT: ascending phase time; PPT: plateau phase time; DPT: decline phase time; MNG: stripping yield; tMBG: overmilking time. ^a,b,c,d,e,f^: Means in the same line with a different superscript letter are significantly different (*p* < 0·05).

## Data Availability

The data used in this study are available on reasonable request from the corresponding author.
